# The Book World of Medicine and Science

**Published:** 1906-01-27

**Authors:** 


### The Book World of Medicine and Science.

A History of English Philanthropy from the Dissolution of the Monasteries to the Taking of the First Census. By B. Kirkman Gray. (London: P. S. King and Son. Pp. 312. Price 7s. 6d. net.)

To adjust wisely philanthropic enterprise to the distress of poverty, it is desirable that both should be studied scientifically and illuminated by the revelations of history. Mr. Kirkman Gray, in his most valuable and interesting study, has furnished us with an important chapter on the evolution of philanthropy as evidenced in this country. Beginning with the overthrow of the monasteries, he carefully traces the development and manifestations of charity during the Elizabethan period, through the seventeenth and eighteenth centuries, and up to the epoch-marking first census at the beginning of the nineteenth century. His object is not so much to indicate the ætiology and pathology of poverty as to record the practical work originated and carried out under the impetus and motive power of the philanthropic idea. By adopting the methods of the student of evolution, he has secured a sound basis and systematic means for the investigation and presentation of his subject, which at the present day is peculiarly opportune. Among so much that is valuable, we may draw particular attention to his excellent description of hospitals in the eighteenth century. Most of the book deals with bygone and forgotten periods, and superficial readers may be inclined to view it as but of historical value, forgetting that a clear understanding of the present can only be obtained by shedding on it the penetrating light of the past. This work is a monument of painstaking research. Every page gives evidence of laborious inquiry, discriminating investigation, and judicial sifting of material. It is a work which should be read, not only by those connected with or interested in hospitals or other philanthropic institutions, but by all those who by public co-operation or by private charity seek to assist the deserving and discourage the unworthy, and thus by the elimination of abuse in all departments of philanthropy, to secure the highest efficiency of each human unit and the elevation of the whole nation. We could have wished that the author had continued his studies through the nineteenth century, and indicated the means and measures by which the ever-present problem of poverty might be approached with permanent advantage by the true and living spirit of philanthropy which has never been more active and desirous of fulfilling its real mission than in these early years of the twentieth century.

